# Diversity of *Toxoplasma gondii* strains at the global level and its determinants

**DOI:** 10.1016/j.fawpar.2019.e00052

**Published:** 2019-04-01

**Authors:** L. Galal, A. Hamidović, M.L. Dardé, M. Mercier

**Affiliations:** a INSERM, Univ. Limoges, CHU Limoges, UMR 1094, Institut d'Epidémiologie et de Neurologie Tropicale, GEIST, 87000 Limoges, France; b Centre National de Référence Toxoplasmose/Toxoplasma Biological Resource Center, CHU Limoges, 87042 Limoges, France

**Keywords:** *Toxoplasma gondii*, Strains, Diversity, Population structure, Evolution determinants

## Abstract

The population structure of *Toxoplasma gondii* is characterized by contrasting geographic patterns of strain diversity at different spatial scales: global, regional and even local scales in some regions. The determinants of this diversity pattern and its possible evolutionary mechanisms are still largely unexplored. This review will focus on three main dichotomies observed in the population structure of the parasite: (1) domestic *versus* wild, (2) South America *versus* the rest of the world and (3) intercontinental clonal lineages *versus* regional or local clonal lineages. Here, the impact in terms of public health of this remarkably contrasting geographic diversity of *T. gondii* populations is discussed, with emphasis on the role of globalization of exchanges that could lead to rapid evolution of *T. gondii* population spatial structure and new challenges in a One Health context.

## Highlights

• Recombination events drive the evolution of population structure of *Toxoplasma gondii.*
• The population structure of *Toxoplasma* is different in wild and domestic environments.
• Virulence of *Toxoplasma* strains in reservoir hosts influences selection of local strains.
• Globalization of exchanges will impact the population structure of the parasite.
• Clinicians should be aware of more pathogenic strains imported from the wild environment or from South America.

The will to understand the determinants of clinical variability observed in different host species, particularly in humans, motivated studies on *Toxoplasma gondii* isolates' diversity with the hypothesis of a role of the infecting strain in this variability. Early work on the genetic diversity of *T. gondii* worldwide studied human or domestic animal isolates mainly from Europe and North America (Darde et al., 1988; Dardé et al., 1992; Sibley and Boothroyd, 1992; Howe and Sibley, 1995). Subsequently, studies based on a more diversified sampling, in terms of host and geographical distribution, have revealed a much more complex genetic diversity of the parasite (Ajzenberg et al., 2004; Lehmann et al., 2004, Lehmann et al., 2006; Khan et al., 2007, Khan et al., 2011b; Su et al., 2012). In addition, the transition from unilocus to multilocus genotyping, more representative of the genome, enabled a more reliable determination of a strain allelic composition and the detection of potential genomic recombinations. The techniques developed for multilocus genotyping relied mainly on the analysis of PCR-restriction fragment length polymorphism (PCR-RFLP) markers or of microsatellite (MS) markers. PCR-RFLP genotyping, relatively inexpensive and without the need of sophisticated equipment, is based on the analysis of 10 markers distributed over 8 chromosomes and one apicoplastic marker (Su et al., 2006; Dubey et al., 2007). A specific number is attributed to each genotype according to the codification adopted by the designation system *ToxoDB* (toxodb.org). Microsatellite markers, more polymorphic, are helpful to trace outbreak and recent events of the evolutionary history of living organisms. The most recent and used technique is based on analysis of 15 MS markers distributed over 11 different chromosomes (Ajzenberg et al., 2010). The designation of clonal lineages characterized by this technique often refers to their geographic origins (for example *Chinese 1* or *Africa 3*). The name “unique genotype” or “atypical” is attributed to strains belonging to none of the clonal lineages defined by this genotyping method.

Using multilocus genes sequencing (MLST) - in which the nucleotide sequences of several loci coding for housekeeping genes are analysed - or Whole Genome Sequencing, this diversity was shown to cluster into 16 haplogroups belonging to six ancestral groups (clades) and distributed throughout the world (Su et al., 2012; Lorenzi et al., 2016). Correspondence between Whole Genome Sequencing (Lorenzi et al., 2016), Multilocus Sequence Typing (MLST; Su et al., 2012), PCR-RFLP (Su et al., 2012; toxodb.org/toxo), and Microsatellites (Ajzenberg et al., 2010) is presented in Table 1 together with the main geographical distribution of the genotypes. While some of these haplogroups are supported by genetically homogeneous strains belonging to well-defined clonal lineages, other haplogroups form much more heterogeneous genetic groups explaining the discrepancies between classifications in haplogroups according to genotyping methods (Table 1). This heterogeneous effect is probably due to recombinant strains that do not show a clear membership in a particular haplogroup. As pointed out by Wendte et al. (2011), we do not know whether these haplogroups represent real phylogenetic entities, or rather points on a continuum that will merge as more isolates are being analysed.

**Table 1:**
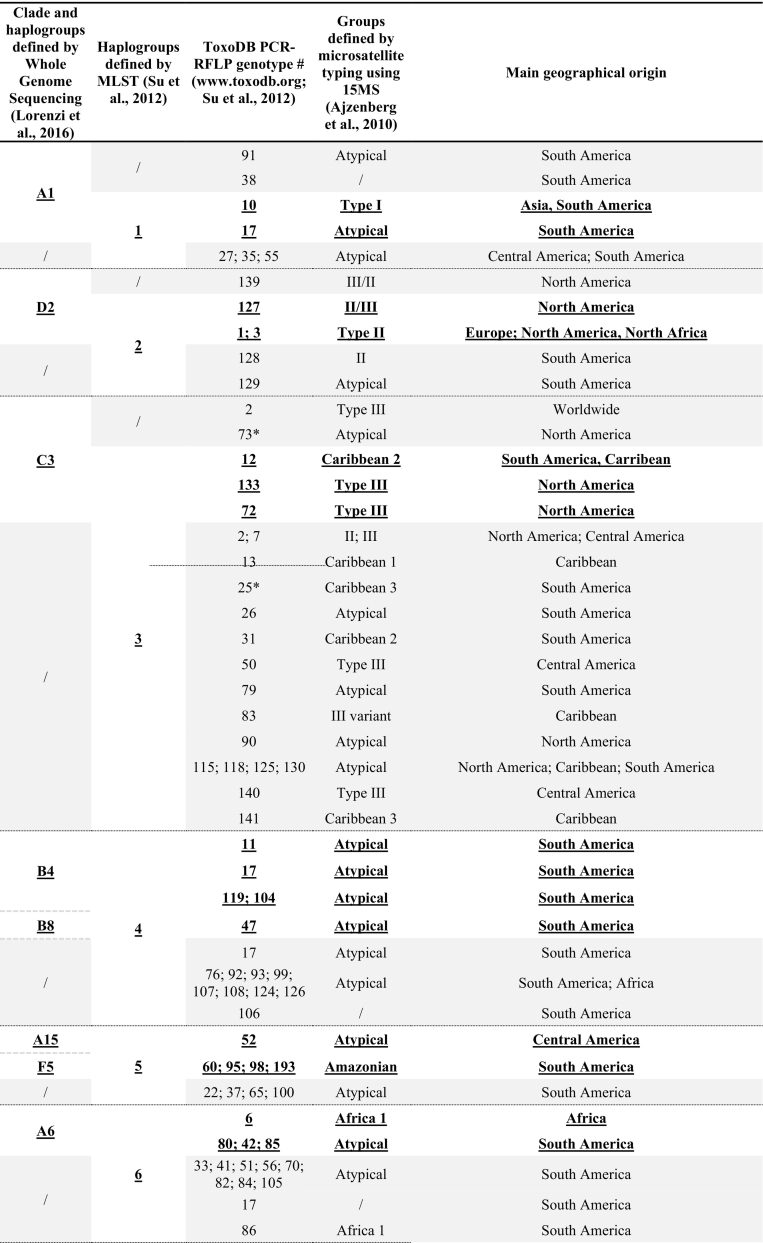

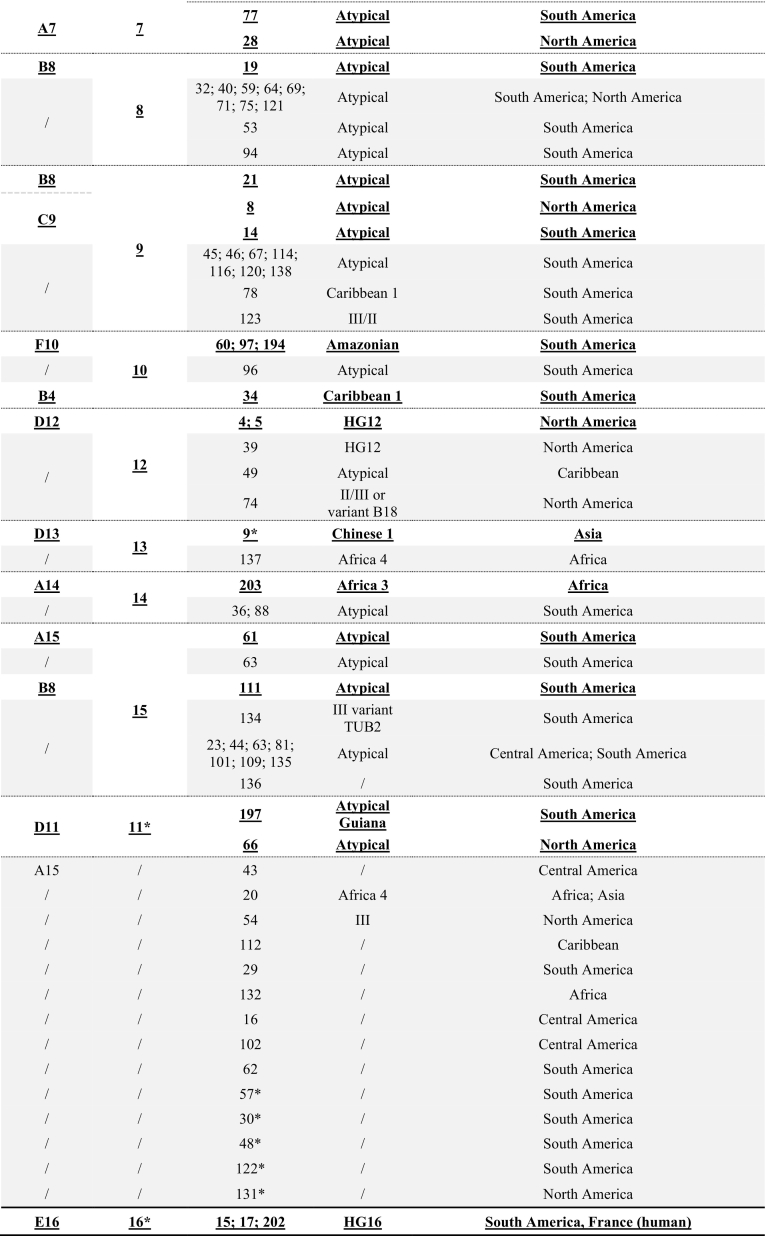
Correspondence of *Toxoplasma gondii* genotypes between Whole Genome Sequencing, MLST, PCR-RFLP or 15 MS markers and their main geographical origin.

/: Haplogroup not defined.

*Grouping did not correspond between Network and Structure analyses (Su et al., 2012).

Bold and underlined: matching information for all analyses.

Multilocus genotyping brought to light a predominantly clonal structure of *T. gondii* populations in most areas of the world. Indeed, it appears that a limited number of predominant intercontinental or regional lineages constitute the bulk of the parasite's diversity. In Europe, type II strains and, to a lesser extent, type III strains, are largely predominant, both in domestic and wild environments (Ajzenberg et al., 2002; Messaritakis et al., 2008; Richomme et al., 2009; Marković et al., 2014; Calero-Bernal et al., 2015; Verma et al., 2015; Klun et al., 2017). In North America, domestic isolates are comparable to those in Europe (types II and III), but strains belonging to haplogroup 12 predominate in the wild environment (Dubey et al., 2011; Khan et al., 2011a; Su et al., 2012; Verma et al., 2015; Jiang et al., 2018). A significant proportion of unique atypical strains have been identified on this continent in wildlife, but also in domestic grazing animals, which are likely to encroach on the home ranges of some wild felids (Pomares et al., 2011; Jiang et al., 2018). In Asia and Africa, although little data exist for the wild environment, genotyped strains have so far been most often classified as belonging to a limited number of intercontinental (types II and III) and regional clonal lineages such as *Chinese 1* in China or *Africa 1* and *Africa 3* in Africa (Ajzenberg et al., 2009; Mercier et al., 2010; Chaichan et al., 2017; Galal et al., 2018a). However, this global trend does not apply to tropical South American countries, where the genetic diversity of the parasite is much higher. In South American domestic environment, even if some local clonal lineages have been identified with some Caribbean and Brazilian strains (Pena et al., 2008; Ajzenberg et al., 2009; Mercier et al., 2011), there is a significant proportion of atypical strains that do not seem to fit into an exclusively clonal propagation pattern, but rather would indicate a frequent incidence of genomic recombinations that generate diversity (Shwab et al., 2014). In the non-anthropized part of the Amazonian forest, which represents an authentically wild environment, strains of *T. gondii* are highly diversified and the population structure of the parasite seems compatible with a panmictic mode of reproduction (Mercier et al., 2011).

In this review, we synthesize the state of art about the evolutionary mechanisms explaining this contrasted diversity in *T. gondii* populations throughout the world. This review is structured around three main dichotomies observed in the population structure of the parasite: (1) domestic *versus* wild, (2) South America *versus* the rest of the world and (3) intercontinental clonal lineages *versus* regional or local clonal lineages.

## 1 The dichotomy domestic *versus* wild

*Toxoplasma gondii* is theoretically able to infect all warm-blooded animal species, which offers it a multitude of ecological niches within wildlife. In the wild environment, this diversity of potential intermediate and definitive hosts would nevertheless subject *T. gondii* to different selection pressures that would lead to a significant diversification of the alleles of this parasite to optimize its adaptation to all available ecological niches (Ajzenberg et al., 2004; Blank and Boyle, 2018). In contrast, the domestic cat and a small number of intermediate hosts constitute all the ecological niches available for *T. gondii* in domestic environments. This reduced range of potential hosts would probably favour the transmission of a limited number of *T. gondii* strains specifically adapted to these domestic hosts (Sibley and Ajioka, 2008; Boothroyd, 2009). In line with this, Mercier et al. (2011) have highlighted in French Guiana a clear genetic separation between the highly divergent and diverse strains that circulate in the Amazonian forest and the clonal strains of the anthropized environment. The same dichotomy seems to exist in North America between the wild and domestic environments, albeit to a lesser degree (Jiang et al., 2018). The occurrence of large populations of wild felids on these two parts of the American continent could be one of the factors that allow the persistence of a sylvatic cycle of *T. gondii*, which was found to be quite distinct from the domestic cycle of the parasite. Indeed, in Europe where wild felid populations and their diversity are very limited (only 3 species *Felis silvestris silvestris*, *Lynx lynx* and *Lynx pardinus*), it is the domestic type II lineage that circulates mainly in both wild and domestic animals (Richomme et al., 2009; Aubert et al., 2010; Blaga et al., 2015). The sylvatic cycles of *T. gondii* in Africa and Asia remain largely unknown.

In addition, Khan et al. (2014) showed that domestic strains of *T. gondii* from all over the world share a highly preserved monomorphic version of chromosome Ia (ChrIa). On the contrary, among wild Amazonian isolates, a high genetic variability of the sequences of this chromosome has been demonstrated. Experimental infections of domestic cats with domestic strains of *T. gondii* (4 strains for 8 cats) and with wild strains (3 strains for 6 cats) have shown that these two categories of strains do not have the same capacity to be transmitted by the domestic cat (Khan et al., 2014). The 4 domestic strains caused oocyst excretion in 6 of the 8 exposed cats (2 strains caused oocyst excretion in only one of the 2 exposed cats) while the 3 wild strains were associated with oocyst excretion in only one of the 6 cats exposed. This monomorphic ChrIa could confer a selective advantage to domestic strains for being disseminated by the domestic cat through oocyst shedding. This adaption could therefore facilitate their spread in the domestic environment and strengthen this dichotomy between a wild cycle and a domestic cycle. Experimental infections conducted on a larger number of cats using a more representative panel of *T. gondii* strains would be necessary to confirm this hypothesis.

The existence of this monomorphic ChrIa in domestic strains from all over the world is an argument that supports a recent common history to all domestic strains of *T. gondii* throughout the world. Current domestic strains would therefore not have emerged independently from the wild in various parts of the world due to a putative preadaptation to transmission by domestic cats as previously proposed by Grigg and Sundar (2009). The most plausible hypothesis is that one or several alleles more advantageous for transmission in the domestic environment would have emerged from the wild at the time of the Neolithic revolution and the advent of agriculture. The expansion of agriculture, the spread of domestic cats, rats and mice would then have allowed the intercontinental spread of strains carrying these alleles (Khan et al., 2011b, Khan et al., 2014). Subsequently, possible hybridizations leading to gene introgression from those invasive to local strains would have contributed to the emergence of unique “regional” strains also adapted to cat transmission. This would explain, despite the genetic proximities between domestic strains around the world, the genetic divergences that exist between the different lineages described to date (Wendte et al., 2010).

## 2 The dichotomy South America *versus* rest of the world

As we have just noted, South America stands out significantly from other continents when it comes to the genetic diversity of *T. gondii*. A substantial diversity of strains has been found on this continent. The most recent common ancestor of current *T. gondii* populations appears to have emerged 1.5 Ma ago in the Amazonian forest (Bertranpetit et al., 2017). This ancient occurrence in this favourable environment would have allowed the diversification of *T. gondii* alleles and the accumulation of mutations by genetic drift (Ajzenberg et al., 2004). A complementary hypothesis that could explain the high genetic diversity of *T. gondii* strains in South America would be a more frequent incidence of sexual reproduction between different strains of *T. gondii*. In the Amazonian forest, where 8 species of wild felids coexist, some have vast territories, and can potentially excrete oocysts over several dozens of kilometers around. This could therefore lead to a spatial overlapping of oocysts excreted by different individuals and therefore belonging to different strains of *T. gondii*. In addition, the climate and rainwater runoff could favour the long-term survival of oocysts in this environment and their spread over long distances. This situation would lead to a high frequency of co-infections (or mixed infections) with strains of different genotypes in intermediate hosts living in this environment, which would result in the excretion of recombinant strains by their feline predators after infection. In addition, experimental studies have shown that a mouse infected or vaccinated with a strain of *T. gondii* does not necessarily develop a cross-immunity that protects it from re-infection with other strains of the parasite. This was shown to be particularly true in the case of superinfection of mice by strains known to be virulent for laboratory mice in primary infections (Elbez-Rubinstein et al., 2009; Jensen et al., 2015). As most South American strains are virulent in laboratory mice, the authors of these studies hypothesized that this mechanism could lead to a high prevalence of co-infection in intermediate hosts of *T. gondii* in South America. This putative pattern could hence lead to more frequent sexual recombinations in the feline hosts that feed on those superinfected intermediate hosts, leading to the emergence of *T. gondii* strains with novel genetic assortment. Nevertheless, studies performed in regions where virulent strains for laboratory mice are frequent (in South America and tropical Africa) show that mixed infections are rare at least among domestic hosts (Dubey et al., 2003, Dubey et al., 2008; Pena et al., 2008; Mercier et al., 2010, Mercier et al., 2011), which seems to contradict this assumption.

In summary, if the high genetic diversity of *T. gondii* in South America could be due to a panmictic reproduction regime, it could also be explained by the existence of a significant number of parasite lineages (Dubey et al., 2007) due to its ancient presence on this continent. This long evolutionary history could have allowed the constitution of a substantial genetic pool through the accumulation of mutations and of sexual recombinations (Wendte et al., 2010).

## 3 The dichotomy intercontinental *versus* local or regional clonal lineages

The modes of migration of *T. gondii* strains remain hypothetic, but the wide range of host species of the parasite suggests a multitude of routes for its short or long-distance migrations. Among those hosts, migratory birds were proposed in a number of past studies (Prestrud et al., 2007, Prestrud et al., 2008; Can et al., 2014; Karakavuk et al., 2018) as possible vectors for the intercontinental spread of parasite strains. Karakavuk et al. (2018) have thus proposed, without being able to demonstrate it, migratory birds as an explanation for the presence in Turkey of strains belonging to the *Africa 1* lineage (Döşkaya et al., 2013). This hypothesis of migratory birds seems plausible in view of the quantitative importance of these flows, which have been going on for millions of years. However, the presence of clearly different strains between, for example, North and South America, although linked by significant migratory bird flows, suggests that this migration pathway plays at most a secondary role in the spread of *T. gondii* (Lehmann et al., 2006). Natural migrations of terrestrial hosts of *T. gondii* could also have a role in the spread of *T. gondii* at local or regional scales, but their role would appear to be minimal in the intercontinental spread of *T. gondii* strains, at least at a reduced time scale. Today, if some parasite lineages exhibit a country-wide or a regional distribution, some lineages have substantially extended their distribution range and have successfully colonized vast areas of the world. This is particularly the case for type II lineage, which is the predominant lineage in North America, Europe and throughout the Mediterranean region (Shwab et al., 2014; Chaichan et al., 2017; Galal et al., 2018a). Type III, although rarely predominant, show an even wider distribution range and is considered as cosmopolitan. Since the emergence of these two lineages during the Neolithic period about 10,000 years ago (Su et al., 2003) in the Middle East, human activities would have contributed to the spread of these two lineages (Shwab et al., 2018). This phenomenon would have taken on a new dimension since the 16th century with the intensification of world trade, which would have allowed the trans and intercontinental spread of these two lineages. In particular, maritime transport, an important vector for the spread of cats, brown rats, black rats and house mice by European vessels (Aplin et al., 2011; Bonhomme et al., 2011) since the slave trade and colonial times, could have allowed the spread of type II and III strains from European ports to ports of other regions involved in these trades (Lehmann et al., 2006; Galal et al., 2018a, Galal et al., 2018b). In addition to being putative vectors of intercontinental migration of *T. gondii* strains, invasive rodents may cause deeper changes in parasite populations in newly colonized areas. Indeed, these small mammals, which are probably the most relevant reservoirs of *T. gondii* in the domestic environment (Dubey et al., 1995; Hejlícek et al., 1997), show variable adaptations to the different parasite lineages. For example, a recent experimental study (Hassan et al., 2018) showed that the different sub-species of *Mus musculus* have highly contrasted responses to certain *T. gondii* strains. It was observed that, unlike *M. m. domesticus* which die in few days when infected by *T. gondii* strains of type I lineage, *M. m. musculus* and *M. m. castaneus* are resistant to these strains. However, wild-derived house mice of all three sub-species develop lethal toxoplasmosis following infection by the African lineage *Africa 1* (FOU strain) and most of the South-American strains. In Africa, the house mouse *M. m. domesticus*, is widely prevailing in the North of the continent for several millennia, a region where the *T. gondii* lineage *Africa 1* is virtually absent (Galal et al., 2018a). In contrast, *M. m. domesticus* has been introduced more recently in tropical regions of Africa (probably during the colonial period) where *Africa 1* is highly prevalent (Dalecky et al., 2015). It can be speculated that the virulence expressed by *Africa 1* toward the invasive house mice could act as an obstacle to the invasion by mice of areas where this parasitic lineage prevails, or on the contrary lead to the decline of this parasitic lineage in areas where this invasive host manages to impose itself and proliferate (Galal et al., 2018b). In more resistant hosts, the heightened virulence for *M. musculus* of some lineages could give the parasite an increased ability for infection and, therefore, give it a selective advantage over other less virulent strains (Khan et al., 2009). In this sense, it has been shown in Africa that some species of small native commensal mammals (*Cricetomys gambianus*, *Crocidura olivieri*) may be competent reservoirs of the *Africa 1* lineage (Galal et al., 2018b). In addition, the native African rodent *Mastomys natalensis* was shown to survive infection by type I lineage which belongs to the same clade as the *Africa 1* lineage (Clade A; Table 1). The absence of virulence of type I in *M. natalensis* would make this rodent another possible competent reservoir for *Africa 1*. Indeed, only strains that successfully cause chronic infection in local rodent species will be likely to be transmitted to cats and contaminate the environment (Lilue et al., 2013). This mechanism could therefore shape *T. gondii* population structures around the world, as the transmission and persistence of *T. gondii* strains would be determined by species-specific profiles of genetic susceptibility and resistance to the different parasitic strains.

## 4 Public health impact

As mentioned in the introduction, one of the objectives of the initial studies on *T. gondii* genetic diversity was to test the hypothesis of a role of the infecting strain in the clinical aspects of toxoplasmosis. At the present time, an increasing number of studies shows the importance of the parasitic strain factor in human toxoplasmosis epidemiology due to the strong geographical contrasts in the diversity of the parasite (Ajzenberg et al., 2004; Khan et al., 2006; Dardé, 2008; Xiao and Yolken, 2015). Thus, the dichotomies we have previously described concerning *T. gondii* diversity can be related with aspects of human toxoplasmosis.

Many cases of multivisceral toxoplasmosis in immunocompetent people have been reported in French Guiana caused by highly pathogenic strains of the parasite from the wild (Carme et al., 2002, Carme et al., 2009). These disseminated forms of toxoplasmosis, also known as “Amazonian toxoplasmosis”, have been diagnosed more often since the practitioners in charge of this type of case were sensitized to this particular form of toxoplasmosis. It would therefore be interesting to explore the existence of this type of severe clinical forms linked to a wild cycle in other well-preserved environments, as in tropical Africa or Asia. In these two regions, biotopes similar to those of the Amazonian region and more widely of South America are found with a large range of definitive and intermediate hosts (savannahs and equatorial forests of Central Africa and Southeast Asia).

Likewise, in South America, there is a high prevalence of severe ocular forms, which can represent a significant public health problem, particularly in certain regions of Brazil, Colombia and northern Argentina (Glasner et al., 1992; Gilbert et al., 2008; de-la-Torre et al., 2013; Rudzinski et al., 2016). These severe forms of ocular toxoplasmosis are at least partly related to pathogenic strains of *T. gondii* circulating throughout South America in association with the genetic diversity hot spot of the parasite on this continent (Carme et al., 2002; Khan et al., 2006; Demar et al., 2007). This relationship should be further investigated in this context. In North America, an association between the atypical strains prevailing on this continent and the incidence of severe ocular and systemic disease in immunocompetent patients has been recently made (Pomares et al., 2018). In Europe, the burden of disease related to toxoplasmosis mainly concerns certain risk groups such as congenitally infected foetuses and immunocompromised individuals, since the strains of the parasite on this continent are not very pathogenic for immunocompetent individuals (Dardé, 2008). Here again, the challenge in the coming years will be to associate the clinical data with the genetic diversity of the parasite for regions still unexplored in this field of human pathogenicity such as Africa and tropical Asia.

Finally, beyond past maritime trade or migratory birds, the current globalization of trade seems to be causing risk situations that pose new research and public health challenges. In France, cases of severe human toxoplasmosis have been reported due to the consumption of imported South and North American horsemeat contaminated with highly pathogenic strains of the parasite (Elbez-Rubinstein et al., 2009; Pomares et al., 2011). In addition to the transport of contaminated meat products, the transport of domestic animals or unintentional transport of *T. gondii* infected pests could contribute to the intercontinental spread of new strains of the parasite, pathogenic or not.
